# The value of a vaginal sample for detecting PAMG-1 (Partosure®) in women with a threatened preterm delivery (the MAPOSURE Study): protocol for a multicenter prospective study

**DOI:** 10.1186/s12884-020-03129-x

**Published:** 2020-08-03

**Authors:** Emilie Marie, Guillaume Ducarme, Marion Boivin, Virginie Badon, Hélène Pelerin, Aurélie Le Thuaut, Zeineb Lamoureux, Valéry-Pierre Riche, Norbert Winer, Thibault Thubert, Vincent Dochez

**Affiliations:** 1 Service de Gynécologie-Obstétrique, CHU de Nantes, Nantes, France; 2 Service de Gynécologie-Obstétrique, CHD Vendée, La Roche sur Yon, France; 3 Centre d’Investigation Clinique CIC FEA, CHU de Nantes, Nantes, France; 4 Unité de Recherche Clinique URC, CHD Vendée, La Roche sur Yon, France; 5 Plateforme de statistiques - Direction de la Recherche CHU de Nantes, Nantes, France; 6 Coordination Cellule Recherche Non Interventionnelle - Direction de la Recherche CHU de Nantes, Nantes, France; 7 Cellule Innovation – Département Partenariat et Innovation - Direction de la Recherche CHU de Nantes, Nantes, France

**Keywords:** preterm birth, PAMG-1, Partosure, cervical length, antenatal corticosteroid, preterm labor

## Abstract

**Background:**

Threatened preterm delivery (TPD) is the leading cause of inpatient admissions during pregnancy. The ability to predict the risk of imminent preterm delivery is thus a major priority in obstetrics. The aim of our study is to assess the diagnostic performance of the test to detect the placental alpha microglobulin 1 (PAMG-1) for the prediction of delivery within 7 days in women with TPD.

**Methods:**

This is a prospective multicenter diagnostic study. Inclusion criteria are singleton pregnancy, gestational age between 24 + 0 and 33 + 6 weeks inclusive, cervical measurement 25 mm or less assessed by transvaginal ultrasound (with or without uterine contractions), clinically intact membranes and cervical dilatation < 3 cm assessed by digital examination.

According to the current protocol, when a women presents with TPD and the diagnosis is confirmed by transvaginal ultrasound, a vaginal sample to test for genital infection is performed. At the same time, the midwife will perform the PartoSure® test. To perform this analysis, a sample of cervicovaginal secretions is taken with the vaginal swab furnished in the test kit.

The primary outcome is the specificity of the PartoSure® test of women who gave birth more than 7 days after their hospitalization for TPD. The secondary outcomes are the sensitivity, PPV, and NPV of the Partosure® test and the factors associated with false positives (with a univariate logistic regression model).

Starting with the hypothesis of an anticipated specificity of 89%, if we want to estimate this specificity with a confidence interval of ± 5%, we will require 151 women who do not give birth within 7 days. We therefore decided to include 400 women over a period of two years to have a larger number of events (deliveries within 7 days).

**Discussion:**

The different tests already used such as fetal fibronectin and phIGFBP-1, are not sufficiently relevant to recommend their use in daily practice. The different studies of PAMG-1 described above thus provide support for the use of this substance, tested by PartoSure®. Nonetheless, other larger studies are necessary to validate its use in daily practice and our study could answer this question.

**Trial registration:**

NCT03401255 (January 15, 2018)

## Background

Threatened preterm delivery (TPD) is the leading cause of inpatient admissions during pregnancy [1]. It is defined by a risk of preterm delivery before 37 weeks of gestation. Its prevalence in France is estimated at 60,000 cases per year [2], which represents about 7.5% of live births[3]. It is complicated by preterm delivery in nearly 30% of cases in singleton pregnancies [4–6].

The principal complication of TPD is preterm birth, which is associated with higher rates of neonatal morbidity and mortality. In 2010, around 15 million children worldwide were born before 37 weeks of gestation, that is, around 11% of live births [1, 7]. In France, approximately 60,000 children (7.4%) are born before 37 weeks of gestation each year, half of them after spontaneous labor [1].

Preterm delivery is the leading factor associated with perinatal morbidity, responsible for neonatal complications such as acute respiratory distress syndrome and necrotizing enterocolitis [8–10]. Neonatal mortality and morbidity are closely associated with gestational age at birth and are highest for fetuses born at the earliest gestational ages [11]. The ability to predict the risk of imminent preterm delivery is thus a major priority in obstetrics.

Although there is currently few treatment options to delay childbirth in the event of a short cervix [12], the ability to discriminate women who will deliver preterm from those who will give birth at term would meet two related objectives: to enable early preventive treatment and to avoid treatment and admissions determined subsequently to be futile and which generate adverse effects, potentially excessive prescriptions of corticosteroids, and futile expenses [2].

Various biochemical markers have been proposed to improve the identification of women at risk of spontaneous preterm delivery, in the hope that their use could reduce its prevalence. Foetal fibronectin and phIGFBP-1 have both shown their effectiveness in ruling out spontaneous preterm delivery within 7 to 14 days, due to their good Negative Predictive Values (NPV), but their low Positive Predictive Values (PPV) mean that they lack precision in identifying the patients at high risk of spontaneous preterm delivery within 7 days [13–15]. For these reasons, numerous hospitals have abandoned its use in daily practice, and its use would require further comparative studies [1].

Recent studies have suggested that the detection of the placental alpha microglobulin 1 (PAMG-1) protein in vaginal secretions by the PartoSure® test among women with TPD symptoms and intact membranes may have good PPV for spontaneous preterm delivery within 7 days [16, 17].

The protein Placenta Alpha Microglobulin-1 (PAMG-1) is found in high concentrations in amniotic fluid. The PartoSure® test is an immunochromatographic test designed to detect PAMG-1 in vaginal secretions when preterm delivery is imminent [18]. This test uses monoclonal antibodies with the sensitivity to detect 4 ng/µL of PAMG-1 in vaginal secretions. This protein is released by decidual cells in the amniotic cavity throughout pregnancy. It is found in vaginal secretions in women with symptoms of labor and for whom delivery is imminent.

According to the study by Lee et al. [18], this mechanism may be explained by the protein’s transudation through the pores of the fetal membranes during uterine contractions, or by the degradation of these membranes’ extracellular matrix due to an inflammatory process during labor or an infection. The PartoSure® test is simple, fast, and noninvasive, in the form of a dipstick (reagent strip) that is read instantaneously.

The test is especially interesting given that the repetition of corticosteroid treatment is currently debated and its effect is most beneficial if delivery takes place in the 24 h to 7 days after its administration [1]. Although some studies show a beneficial effect of the repetition of cures in the first weeks of life [19], repetition of corticosteroid treatment could be responsible of an increase in insulin resistance and cardiovascular risk in adulthood, as well as obesity [20]. In addition, repeated courses of corticosteroids do not seem to have any benefit on morbidity or mortality at the age of 5 years [21]. In 2016, the French National College of Gynecologists and Obstetricians (CNGOF) mentioned the use of the PAMG-1 test, but more studies are necessary to reach a conclusion about its use [1]. Further studies appear essential to specify in more detail the value of this test in women with TPD.

The aim of our study is to assess the diagnostic performance of the test to detect PAMG-1 for the prediction of delivery within 7 days in women with TPD.

## Methods / Design

### Protocol version

The reference protocol is RC17_0247 – Etude Maposure.

The N° ID RCB is: 2017-A02715-48.

The protocol version is n°9 (20/11/2017).

### Trial design

This is a prospective multicenter diagnostic study.

This is a non-interventional study. There was therefore no written consent necessary but a recipe for no opposition.

### Participating centers

The two participating centers are the Nantes University Hospital Center (UHC) (Nantes, France) and the Vendée District Hospital Center (La Roche sur Yon, France).

### Eligibility criteria

Patients are recruited from among the pregnant women receiving prenatal care in both centers.

#### Inclusion criteria

- Adult woman (at least 18 year old), legally competent.
- provides oral consent after information.
- singleton pregnancy.
- affiliated with the French CNAMTS (salaried workers’ health insurance).
- gestational age between 24^+ 0^ and 33^+ 6^ weeks inclusive.
- cervical measurement ≤ 25 mm assessed by transvaginal ultrasound, with or without uterine contractions.
- clinically intact membranes.
- cervical dilatation < 3 cm assessed by digital examination.

The threshold of 25 mm for cervical measurement would be relevant to rule out the risk of spontaneous premature delivery at 48 h and 7 days [1]. It seems essential to us not to miss a patient who risks giving birth in the hours or days to come, taking into account the consequences of a premature child who has not received corticosteroid therapy. We therefore include more patients than if we took the 15 or 20 mm threshold (ultrasound plays the role of sensitivity) [22], hence the interest of a specific test.

#### Exclusion criteria

- presence of vaginal bleeding.
- history of cervical conization.
- history of uterine malformation (bicornuate bicervical uterus, bicornuate unicervical uterus, unicornuate uterus, septate uterus).
- presence of polyhydramnios with amniotic fluid index > 25.
- obvious premature rupture of the membranes.
- clinical or laboratory-confirmed chorioamnionitis.
- cervical cerclage.
- intercurrent obstetric disease that might induce preterm birth.
- patient hospitalized for more than 24 h in another hospital or department for TPD.

We excluded patients who had a conization because the cervix is automatically shorter physiologically [23]. The 25 mm threshold cannot then apply and it remains dependent on the presence or not of uterine contractions [24]. However, these patients, like twin pregnancies, are at greater risk of prematurity, and a specific assessment would be necessary.

### Materials

To perform the PartoSure® test, a sample of cervicovaginal secretions is taken with the vaginal swab furnished in the test kit. After removing the swab from its packaging, it is held in the middle of the stem, and its extremity is carefully and gently introduced into the vagina for a length of 5–7 cm (a speculum is not necessary to take this sample), when the woman is in a supine position. The swab should be withdrawn from the vagina after 30 s. Once it has been withdrawn, the swab end containing the sample should be immediately placed into the vial of solvent furnished for this purpose, then rinsed by being rotated several times for 30 s. The swab should then be withdrawn from the vial and discarded as biomedical waste. After the dipstick is removed from its packaging, its white portion (indicated by arrows pointed downward) is inserted into the vial of solvent. The dipstick should be removed from the vial once two bands are clearly visible in the test zone, or after 5 min. Then the results are read on a clean, dry, flat surface. Two bands in the test zone indicate a positive result, while a line in the test zone indicates a negative result. It is not recommended that the results be read or interpreted after the dipstick has been immersed in the vial for more than 10 min. It is important to know that the intensity of the bands can vary. The test is considered positive even if the bands are light-colored or appear shadowed. A result is negative when only the control line appears. A test is considered invalid when the control line is absent.

This test can be used after a vaginal examination, and no special equipment or training is necessary.

It can be used between 20^+ 0^ and 36^+ 6^ weeks of gestation.

According to the current protocol, when a women presents with TPD and the diagnosis is confirmed by transvaginal ultrasound (with a cervical measurement ≤ 25 mm), a work-up to test for infection is performed, comprising a blood sample with a complete blood count (CBC), C-reactive protein (CRP), a urinary reagent strip, and a vaginal sample to test for genital infection. At the same time, the midwife will perform the PartoSure® test. The test will then be discarded, and the result reported in the CRF. Because this vaginal sample is taken at the same time as that for bacteriological testing, there is no additional gynecologic examination and no psychological risk for the woman. This research is therefore classified as noninterventional.

Cardiotocography is performed to verify fetal status and to show uterine contractions.

In case of uterine contractions, if tocolysis was necessary, the patient received either nifedipine (oral administration) or atosiban (intravenous administration), for a maximum of 48 h. Prenatal corticosteroid treatment aimed at fetal pulmonary maturation was performed with betamethasone 12 mg intramuscularly once a day for 2 days.

### Outcome measures

#### Primary outcome

The specificity of the PartoSure® test will be estimated with its 95% confidence interval. It will be defined as the number of women with a negative test divided by the number of women who gave birth more than 7 days after their hospitalization for TPD.

#### Secondary outcomes

- The sensitivity, PPV, and NPV of the Partosure® test for the prediction of delivery within 7 days will be estimated with their 95% confidence intervals.

Sensitivity will be calculated by dividing the number of women who gave birth within 7 days and tested positive (true positives) among all the women who gave birth within 7 days.

The negative predictive value will be calculated by dividing the number of women who did not give birth within 7 days and tested negative (true negatives) by all the women who tested negative. The positive predictive value will be calculated by dividing the number of women who gave birth within 7 days and tested positive (true positives) among all the women who tested positive.

- The same estimates will be performed for the diagnostic capacity of the Partosure® test for predicting delivery within 48 h.
- If the number of individuals allows, the same analyses will be performed according to subgroups for cervical length and gestational age at inclusion.
- The factors associated with false positives will be sought from a univariate logistic regression model. All of the variables significant with a P-value < 0.20 will be introduced into a multivariate model. A backward stepwise procedure will be used to determine the factors associated with a P value < 0.05, which will be retained in the final model.
- Costs will be presented as means with their 95% confidence intervals.

A univariate sensitivity analysis will be performed and will consider the variables of interest identified during the study, such as the cost of the PartoSure® test.

From an economic point of view, women hospitalized in the Homogeneous Patient Group 14Z16Z = False labor and threatened preterm delivery are charged a flat fee of €1,082.06 when she is hospitalized for 1 to 4 days, to which is added a daily charge of €359.21 after the 4th day. These charges must make it possible to cover all healthcare utilization (drugs, medical devices, imaging) generated by the woman during her hospitalization but also the time spent by hospital staff and the hospital’s logistics and general management.

A national accounting database estimated that the mean cost of the management described above was €2,020 in 2015. The Nantes UHC estimated this mean cost in 2016 at €2,592.15. For the women hospitalized at the District Hospital Center of La Roche sur Yon, the estimated cost was €654.26 per day of hospitalization.

An isolated analysis of the price of the drugs used in association with TPD shows that:

- one ampule of magnesium sulfate costs €0.16.
- one ampule of betamethasone costs €0.96.
- one ampule of tractocile costs € 43.90 for the loading dose and €14.29 for the maintenance doses.
- the nifepidine protocol including 4 tablets costs €0.20.

The cost of the PartoSure® test is €56. It will be fully covered by the Natech® pharmaceutical company in our study.

Based on the standard (reimbursed) price for transport by ambulance, the cost of an in utero transfer between two hospital centers includes a flat fee of €51.30 associated with a price of €2.19 per kilometer traveled. When the transfer takes place in the same city, the flat fee of €57.37 is applied without any charge per kilometer.

There is no data monitoring committee because it is a prospective multicenter diagnostic study and non-interventional study.

This research is classified as noninterventional. It was a verbal consent from the patients. The “Patient Protection Committee (CPP) of Paris VIII” approved this protocol on 07/11/2017 as reference RC17_0247. The study will be conducted in compliance with the current approved version of the protocol.

### Sample size

We wish to assess the specificity of the PartoSure® test to see if it diminishes the number of false positives: the sensitivity of the TPD diagnosis is determined by cervical ultrasound, an examination with mediocre specificity.

Starting with the hypothesis of an anticipated specificity of 89% [16, 17], if we want to estimate this specificity with a confidence interval of ± 5%, we will require 151 women who do not give birth within 7 days. We have estimated the prevalence of the event at 10%, representing approximately 17 women. Accordingly a sample size of 168 women is necessary.

Nonetheless, that means that we will have very few events, since the secondary endpoint assesses the test’s sensitivity. The confidence interval will therefore be very wide, because with an estimated sensitivity of 75%, it will be ± 20%.

In 2016, 220 women were hospitalized for TPD between 24 and 34 weeks at Nantes UHC and 50 at the Vendée CHD (data extracted from the hospitals’ statistics departments).

We therefore decided to include 400 women over a period of two years to have a larger number of events (deliveries within 7 days).

The analysis sub-groups could be:

- cervical measurement on transvaginal ultrasound: ≤ 15 mm, 16–20 mm, 21–25 mm.
- gestational age between: 24^+ 0^ and 27^+ 6^ weeks, 28^+ 0^ and 31^+ 6^ weeks, 32^+ 0^ and 33^+ 6^ weeks.

### Statistical methods

Statistical analyses will be performed with SAS v.9.4 software. The primary analysis will be based on the intention-to-treat principle and will be complemented by a per-protocol analysis.

Descriptive analyses will be performed for all of the variables collected and point estimates by 95% confidence intervals for the qualitative and quantitative variables. If the distributions are not normal, medians and interquartile intervals of the corresponding variables will be furnished.

The alpha risk selected is 5%.

## Discussion

Ehsanipoor et al. showed that the presence of PAMG-1, detected by the AmniSure® test, is associated with a high risk of delivery within 7 days when cervical length is less than 20 mm, with a sensitivity of 84% and a specificity of 82% [25]. The AmniSure® test works within a wide range of PAMG-1 concentrations potentially found in vaginal discharge (from 5 ng/ml to 200 µg/ml). The diagnostic accuracy of the test allows it to detect even a minuscule amount of released amniotic fluid. With intact fetal membranes, the test does not normally detect PAMG-1 due to its low background concentration. PartoSure® test detects trace amounts of human PAMG-1 at lower thresholds than AmniSure® test notably by a different vaginal swab: 1 ng/ml.

Another study assessed the effectiveness of the PartoSure® test in detecting PAMG-1 to predict the time until spontaneous preterm delivery in women with symptoms of TPD with intact membranes and cervical dilation less than 3 cm [16]. Its sensitivity for predicting delivery within 7 days was 90% (95% CI 68.3–98.8), its specificity 93.8% (95% CI 86.2–98.0), its NPV 97.4% (95% CI 91.0-99.7), and its PPV 78.3% (95% CI 56.3–92.5).

Transvaginal ultrasound measurement of cervical length is useful for estimating the risk of spontaneous preterm delivery in the general population [22]. In 2010, the French Health Authority (HAS) evaluated the performance of cervical length measurement for the prediction of delivery within 48 h and within 7 days as a function of the threshold selected — 25 mm [2]. In this context, there are even training programs for health professionals, to ensure quality ultrasound images [26, 27]. Although the sensitivity of cervical ultrasound makes it possible to isolate a group of women at risk, 80% of them will not give birth within 7 days [28]. The different tests already used such as fetal fibronectin and phIGFBP-1, are not sufficiently relevant to recommend their use in daily practice. They have good sensitivity (but which is already achieved by transvaginal ultrasound), but low specificity (< 80%) [29]. Furthermore, NPV is dependent on the frequency of preterm birth in the population and can therefore be used less routinely. The different studies of PAMG-1 described above thus provide support for the use of this substance, tested by PartoSure® for the management and diagnosis of TPD. Nonetheless, other larger studies are necessary to validate its use in daily practice and our study could answer this question.

## Data Availability

Not applicable.

## Abbreviations

CPP: Patient Protection Committee
NPV: Negative predictive value
PAMG-1: Placenta Alpha Microglobulin-1
PPV: Positive predictive value
TPD: Threatened preterm delivery
UHC: University Hospital Center
